# The problem with defining foreign birth as a risk factor in tuberculosis epidemiology studies

**DOI:** 10.1371/journal.pone.0216271

**Published:** 2019-04-30

**Authors:** Jennifer L. Guthrie, Lisa A. Ronald, Victoria J. Cook, James Johnston, Jennifer L. Gardy

**Affiliations:** 1 School of Population and Public Health, University of British Columbia, Vancouver, Canada; 2 British Columbia Centre for Disease Control, Vancouver, Canada; 3 Department of Medicine, University of British Columbia, Vancouver, Canada; St Petersburg Pasteur Institute, RUSSIAN FEDERATION

## Abstract

**Objective:**

To examine how stratifying persons born outside Canada according to tuberculosis (TB) incidence in their birth country and other demographic factors refines our understanding of TB epidemiology and local TB transmission.

**Background:**

Population-level TB surveillance programs and research studies in low incidence settings often report all persons born outside the country in which the study is conducted as “foreign-born”–a single label for a highly diverse population with variable TB risks. This may mask important TB epidemiologic trends and not accurately reflect local transmission patterns.

**Methods:**

We used population-level data from two large cohorts in British Columbia (BC), Canada: an immigration cohort (*n* = 337,492 permanent residents to BC) and a genotyping cohort (*n* = 2290 culture-confirmed active TB cases). We stratified active TB case counts, incidence rates, and genotypic clustering (an indicator of TB transmission) in BC by birth country TB incidence, age at immigration, and years since arrival.

**Results:**

Persons from high-incidence countries had a 12-fold higher TB incidence than those emigrating from low-incidence settings. Estimates of local transmission, as captured by genotyping, versus reactivation of latent TB infection acquired outside Canada varied when data were stratified by birthplace TB incidence, as did patient-level characteristics of individuals in each group, such as age and years between immigration and diagnosis.

**Conclusion:**

Categorizing persons beyond simply “foreign-born”, particularly in the context of TB epidemiologic and molecular data, is needed for a more accurate understanding of TB rates and patterns of transmission.

## Introduction

Population-level tuberculosis (TB) surveillance programs and research studies frequently report all persons born outside the country in which the study is conducted under a single label–typically “foreign-born”–without stratification based on birth country incidence or age at immigration [1–5]. In these studies, a person who immigrated from a low TB incidence country (<10 cases/100,000 population) [6] as an infant, and an adult recently arrived from a high-incidence country would be similarly classified, despite likely having very different TB exposure histories.

Similar to other low-incidence settings, TB rates in Canada are considered to be largely driven by reactivation of latent TB infection (LTBI) acquired prior to immigration, with active TB disease most frequently diagnosed within the first two years after immigration [7,8]. Furthermore, 70% of active TB cases are diagnosed in persons born outside of Canada, with the majority of cases occurring in people born in high incidence countries [9]. Accordingly, most low incidence countries focus on targeted screening of persons emigrating from countries with high TB incidence rates [10–12], with thresholds ranging from >15 cases to >100 per 100,000 [13]. In Canada, this threshold is defined as ≥30 cases per 100,000 population [14]. Therefore, focusing on identifying TB among “foreign-born” populations in low incidence settings such as Canada may be appropriate in the scenario of immigration screening, given that the time of immigration is a defined contact point when targeted interventions can be initiated. However, the categorization of large groups of people as “foreign-born” may mask important TB epidemiologic trends and not accurately reflect local transmission patterns.

The objective of the following study was to examine how more detailed reporting of immigration-related demographics, particularly TB incidence in country of birth, years since immigration, and age at immigration refines our understanding of TB epidemiology and local TB spread. For this study, we leveraged data from two population-level datasets [15,16]–to our knowledge the largest TB epidemiologic databases in Canada–allowing for in-depth analyses of both LTBI reactivation and locally transmitted TB cases in the province of British Columbia (BC). This is important for developing effective TB prevention strategies and public health efforts in low-incidence, high migrant-receiving settings.

## Methods

### Study population and design

This study is part of a large initiative aimed at reducing the incidence of active TB in BC, through both the identification of high-risk populations to improve LTBI screening and treatment, and prevention of transmission in the province [17]. We have previously published results from a study focused on identifying populations who are at highest risk of developing active TB after immigration to BC, based on demographic characteristics at the time of arrival [16]. To address the issue of transmission within BC, we also recently published a 10-year retrospective study using 24-locus Mycobacterial Interspersed Repetitive Units-Variable Number of Tandem Repeats (MIRU-VNTR) genotyping [15]. The datasets arising from these two studies provided an opportunity to extend our analyses to examine how stratifying persons born outside Canada according to immigration-related demographics is important for our understanding of TB epidemiology and transmission, which we report here.

#### Immigration cohort

Briefly, in the first cohort, we linked records from the BC Provincial TB Registry [18] to data for from the Immigration, Refugees and Citizenship Canada (IRCC) Permanent Residents database and Population Data BC health administrative databases [19,20]. The databases and linkage methods have been described previously [16]. The cohort for this study included all individuals (*n* = 337,492) who immigrated to Canada and became BC residents between 2005 and 2012.

#### Genotyping cohort

Transmission between individuals in most low-incidence countries is established through a combination of contact investigation and genotyping of *Mycobacterium tuberculosis* (*Mtb*) isolates obtained from culture-positive cases [21–23]. Genotypic clustering may indicate local transmission; however, interpretation of clustering and likely direction of transmission relies on knowledge gained through molecular epidemiology studies that integrate demographic characteristics, such as country of birth, time since immigration, age, and social/behavioural risk factors, with genotyping results [15,24,25]. Therefore, separately, in a second cohort, we extracted individual-level clinical, demographic and risk factor (e.g. HIV, substance use) data from the BC Provincial TB registry [18] and linked these to genotypic data (24-locus MIRU-VNTR) representing 99.3% of all culture-positive TB cases in BC, 2005–2014, as previously described [15]. It should be noted that this cohort included foreign-born visitors, temporary and permanent residents as well as Canadian-born residents, whereas due to the nature of the available databases, the immigration population cohort included only permanent residents.

### Statistical analysis

Birthplace TB incidence rates were derived from yearly country-level incidence data (all forms of TB/100,000 population) [26]. Incidence categories were defined as medium-to-low (<30 cases/100,000), and high (≥30 cases/100,000). Within the immigration cohort, TB rates according to years since immigration, age at time of immigration, and TB incidence in birth country, were calculated as the number of TB cases per 100,000 person-years of follow-up time in BC. Within the genotyping cohort, active TB case counts were summarized stratified by TB incidence in country of birth and represented graphically, with trends over time evaluated by linear regression. We categorized individuals has having arrived <10 years or ≥10 years prior to TB diagnosis based on a recent study indicating differences between these groups [27], and our recent publication suggesting that TB rates level off after approximately 10 years post-immigration [16]. Large genotype clusters were defined arbitrarily as ≥10 cases. We compared demographics and genotypic clustering of individuals between groups using summary statistics including median, interquartile range (IQR) and Mann–Whitney U tests, where appropriate. Analyses were conducted in SAS (v 9.3) and R (v3.4.1).

Ethics approval was granted by the University of British Columbia (certificates #H16–00265, #H12-00910). Patient consent was not required as data was collected as part of routine surveillance and public health activities.

## Results

### Tuberculosis incidence rates and case counts

We analyzed TB incidence rates among 337,492 permanent residents arriving in BC from 2005 through 2012 and found the overall rate in persons from high-incidence countries (27.8/100,000 person-years) was 12-fold higher than in those born in a medium-to-low incidence country (2.3/100,000 person-years). Furthermore, TB incidence rates in BC remained higher among residents from high-TB incidence countries for at least five years after arrival with those arriving from high-incidence countries having an incidence of 40.0/100,000 person-years compared to individuals from medium-to-low countries with less than 1 active TB case per 100,000 person-years (data not shown).

**Fig 1:**
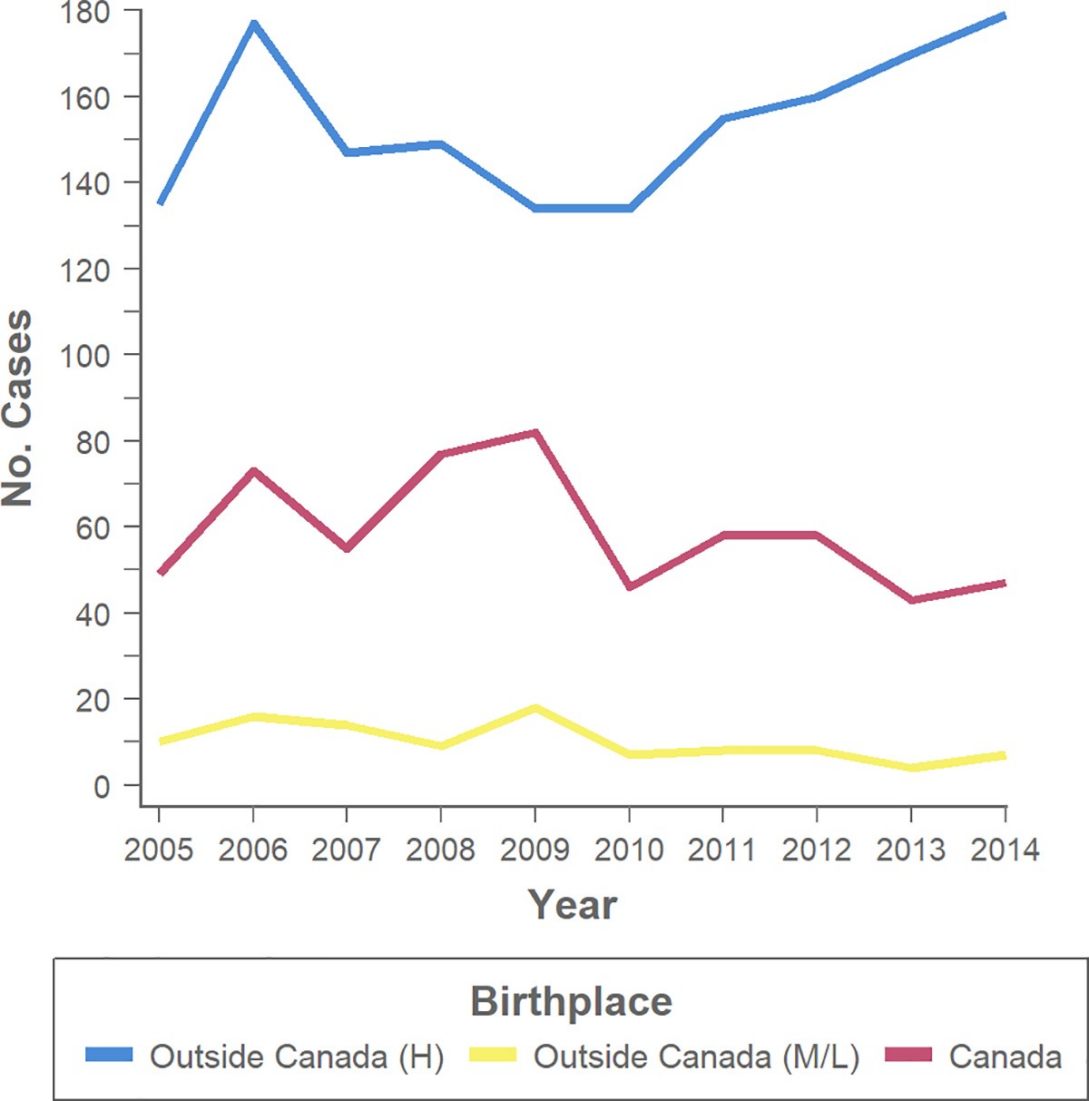
Trends in active tuberculosis diagnoses in British Columbia, Canada. Number of culture-confirmed cases over a 10-year period categorized by birthplace: Outside Canada (H—high-incidence countries [≥30 per 100,000], M/L—medium-to-low-incidence countries [<30 per 100,000]), Canada.

Case counts in the genotyping cohort reflected a similar pattern–amongst culture-confirmed TB diagnoses in which birthplace was known (*n* = 2,229), the number of cases was lowest in persons born outside Canada in regions with medium-to-low TB incidence (Fig 1). While case counts over time slightly decreased in this medium-to-low TB incidence group (*p* = 0.047) and were stable in the Canadian-born group (*p* = 0.246), after 2010, they increased in persons from high-incidence countries (*p* = 0.005).

### Age and time since arrival varies across immigrant groups

**Fig 2:**
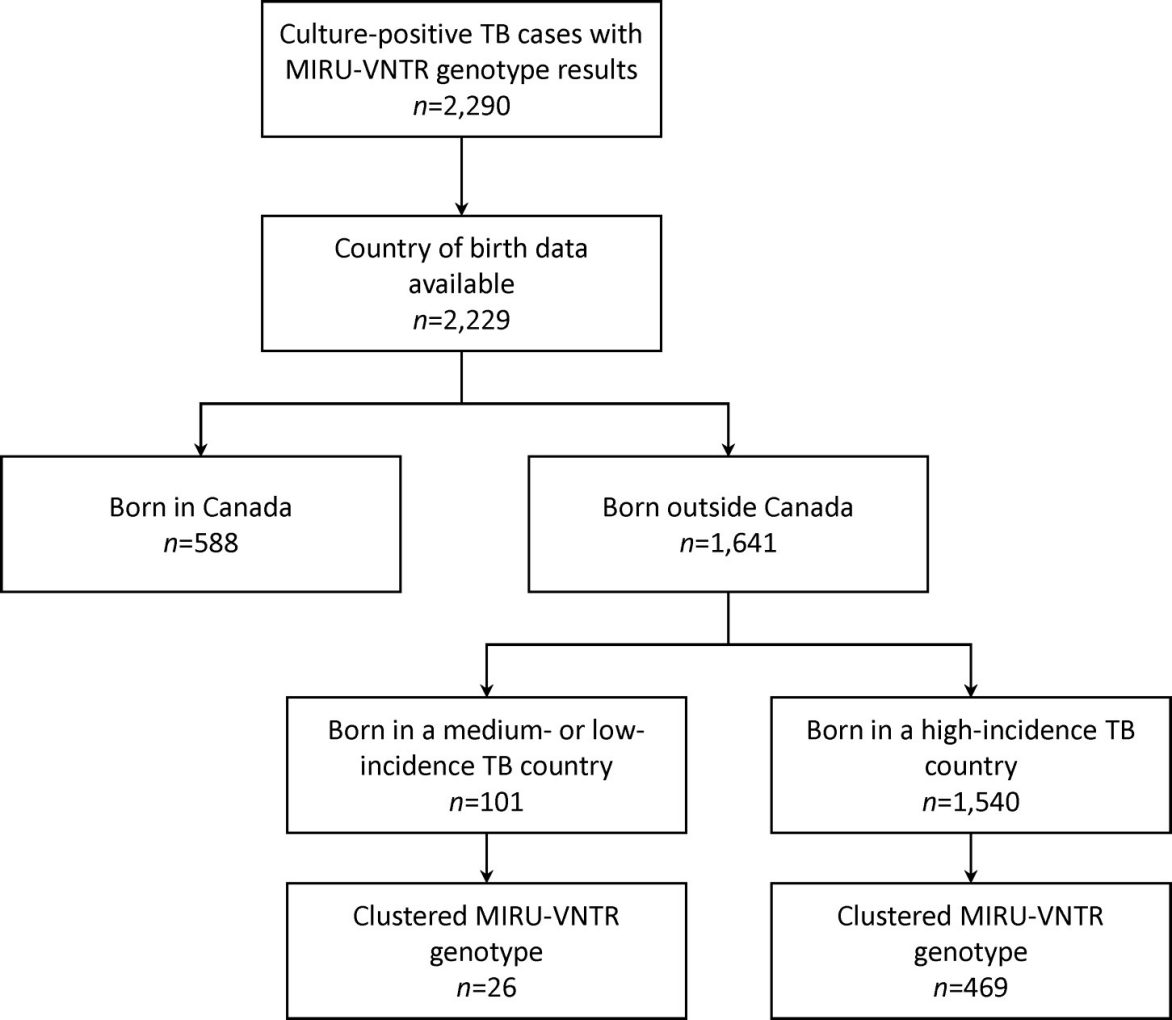
Flow diagram. Culture-positive tuberculosis cases in British Columbia, Canada (2005–2014).

We next examined the various sub-populations with active TB using the genotyping cohort. Our study sample consisted of 2,229 genotyped isolates for which country of birth was known. The majority of persons born outside Canada were from a high-incidence TB country (93.8%, *n* = 1,540), compared to 6.2% (*n* = 101) that were from medium- or low-incidence TB countries (Fig 2). Categorizing each incidence group by time from immigration to diagnosis–within 10 years and ≥10 years–revealed demographic and genotype clustering differences (Table 1). Notably, individuals from medium-to-low incidence countries arriving in Canada ≥10 years prior to diagnosis were distinct. This group had a significantly lower (*p*<0.01) age at immigration with a median age of 28 years (IQR: 12–35), and considerably higher (*p*<0.001) number of years since immigration (median: 39 years, IQR: 23–53) compared to all others in which the median age at immigration ranged from 33–41 and median years since immigration 3–19. Age at diagnosis appeared to trend towards older ages for persons having arrived in Canada ≥10 years prior with median ages of 67 years (IQR: 49–78) and 72 years (IQR: 54–79) for persons from high-incidence and medium-to-low incidence countries, respectively. This is considerably higher than the median ages seen for those having emigrated less than 10 years prior to diagnosis, 36 (IQR: 26–51) and 42 (IQR: 29–55).

**Table 1:** Characteristics of persons born outside Canada in high-incidence (≥30 per 100,000) and medium-to-low incidence (<30 per 100,000) tuberculosis countries according time to diagnosis after arrival category*, British Columbia, Canada (2005–2014).

Characteristic	Diagnosed <10 years after arrival		Diagnosed ≥10 years after arrival	
	High-incidence	Medium- to low-incidence	High-incidence	Medium- to low-incidence
Total—*n*	644	15	855	81
Demographics				
Age at immigration—median (IQR)	33 (24–47)	37 (26–50)	41 (27–57)	28 (12–35)
Age at diagnosis—median (IQR)	36 (26–51)	42 (29–55)	67 (49–78)	72 (54–79)
Years since immigration—median (IQR)	3 (1–5)	3 (0–6)	19 (14–28)	39 (23–53)
Cases with ≥1 risk factor† —*n*(%)	21 (3.7)	2 (16.7)	29 (4.2)	7 (11.9)
Clustering				
Clustered—*n*(%)‡	205 (31.8)	4 (26.7)	249 (29.1)	22 (27.2)
Canadian-born cluster—*n*(%)§	2 (1.0)	1 (25.0)	9 (3.7)	15 (75.0)

Abbreviations: IQR, interquartile range.

*Year of immigration unavailable (*n* = 52).

†Risk factors: HIV positive, illicit drug use, and alcohol misuse. Risk factor data unavailable (*n* = 271).

‡Cluster: ≥ 2 patients that share an identical genotype (24-locus MIRU-VNTR).

§Cluster with >50% Canadian-born individuals; denominator is the number of isolates clustered for each incidence category.

### Tuberculosis transmission varies according to incidence in country of birth

**Fig 3:**
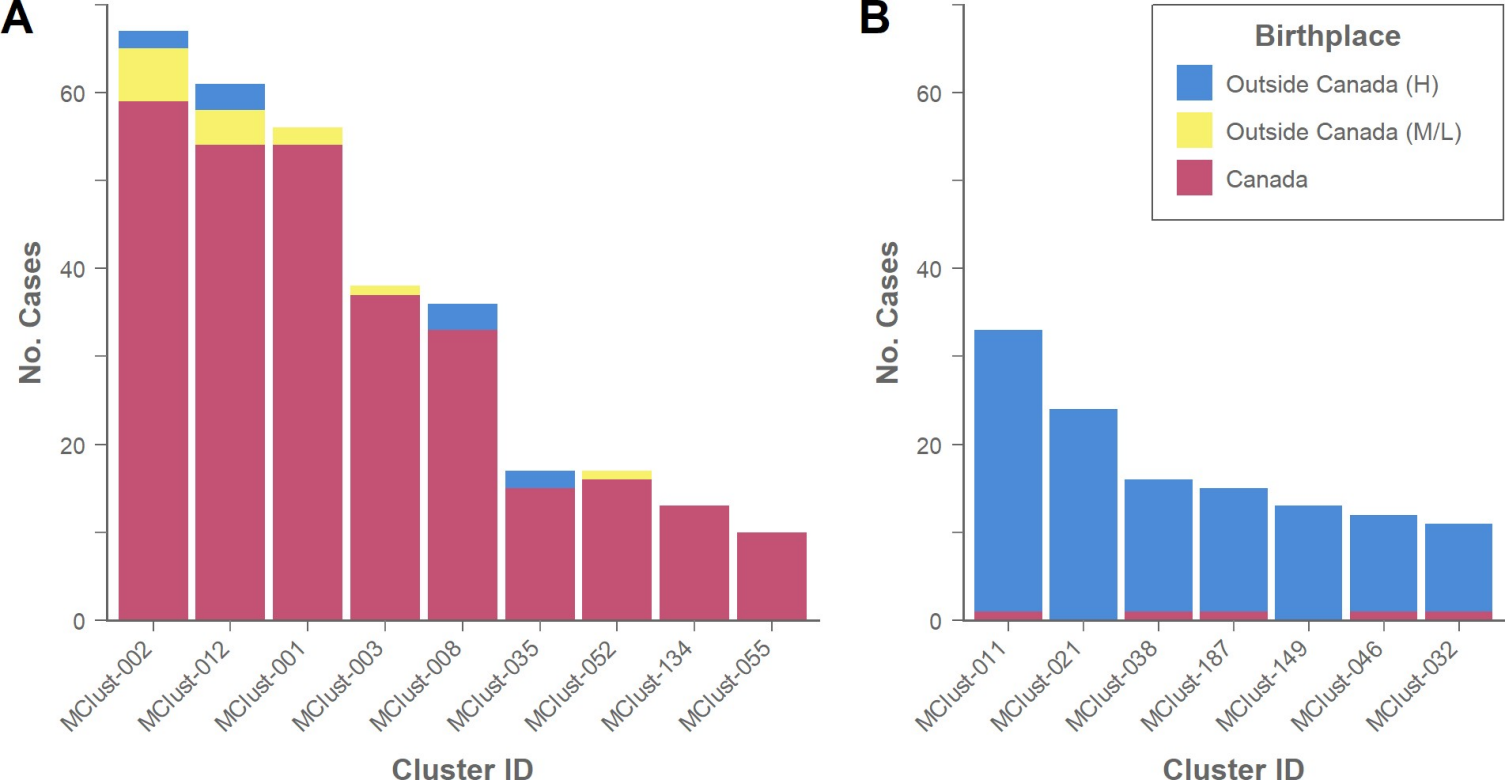
Tuberculosis frequencies by birthplace incidence in large genotypic clusters. The number of tuberculosis cases for each large (≥10 persons) genotypic cluster in British Columbia, Canada (2005–2014) by cluster type: (A) predominantly Canadian-born and presumed to represent local transmission; (B) predominantly persons born outside Canada and presumed to largely represent reactivation of LTBI. Coloured to indicate persons born outside Canada (H—high-incidence countries [≥30 per 100,000], M/L—medium-to-low incidence countries [<30 per 100,000]), or Canadian-born.

Of the 1,641 persons born outside Canada 69.8% (*n* = 1,146) grew a TB isolate with a unique genotype, suggestive of LTBI reactivation. For genotypic clustering the proportion of individuals with *Mtb* isolates belonging to a cluster was comparable between those born outside Canada in a medium-to-low incidence country (25.7%) and those born in a high-incidence country (30.5%), Fig 2. Despite this similarity, closer examination of these groups revealed a number of differences. First, the proportion of cases with *Mtb* isolates genotypically clustered with Canadian-born persons was largest for those from medium-to-low incidence countries, and 87.5% (14/16) of these belonged to large clusters (≥10 cases) consisting of predominantly Canadian-born persons and representing known local transmission (Fig 3). Second, nine of 71 (12.7%) persons born in medium-to-low incidence countries with available data reported risk factors, including HIV and substance use, and seven of these individuals had *Mtb* isolates that genotypically matched isolates from Canadian-born persons. This suggests that these individuals acquired their infection in BC related to risk factors other than birth outside Canada. In contrast, among large genotypic clusters consisting of predominantly foreign-born persons within which individuals were from similar regions of the world e.g. MClust-149 consists of persons from different countries in southern Asia (Fig 3)–clusters that epidemiologic field work suggests largely arose from LTBI reactivation of strains circulating overseas, all of these 119 (100%) people emigrated from high-incidence TB countries, and none reported HIV or substance use. Overall, only 3.3% (50/1499) of persons born in high-incidence countries reported such risk factors.

**Fig 4:**
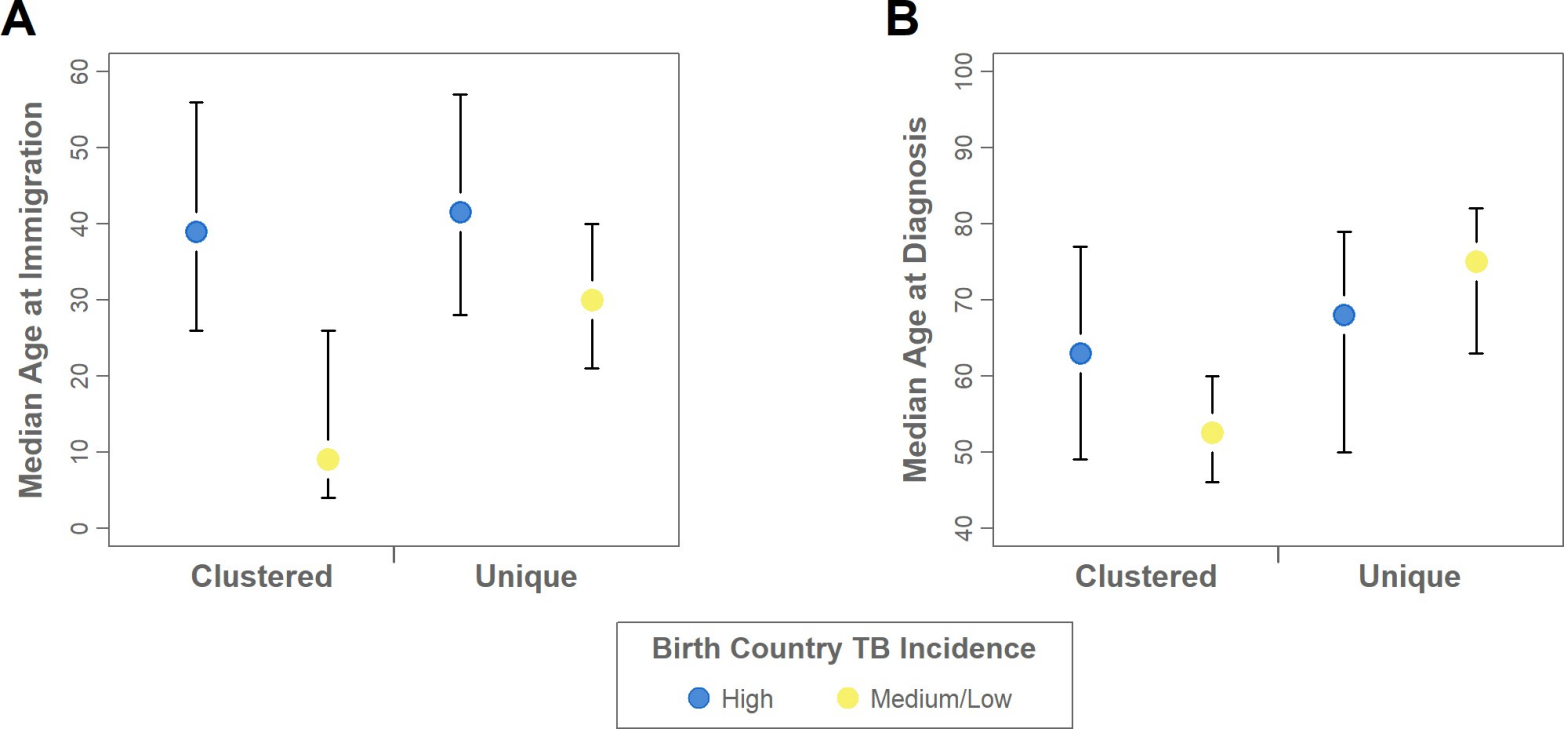
Age and genotype clustering characteristics of immigrants with ≥10 years in Canada. Median and interquartile range, coloured to indicate persons born outside Canada in high-incidence tuberculosis countries (≥30 per 100,000), and medium-to-low incidence tuberculosis countries (<30 per 100,000). (A) Age at immigration. (B) Age at tuberculosis diagnosis.

Shifting focus to the characteristics of persons with ≥10 years in Canada (S1 Table), 77.3% (17/22) of those from medium-to-low incidence countries with genotypically clustered isolates were male and had a considerably lower median age at immigration of 9 years (IQR: 4–26, *p*<0.001) and lower median age at diagnosis of 53 years (IQR: 46–60, *p*<0.01) compared to all other groups (Fig 4). Within this group 68.2% (15/22) were born in the United States or Europe (exclusive of Eastern Europe). Regardless of clustering status persons born in medium-to-low incidence countries had arrived in Canada earlier (median year: 1968, IQR: 1957–1986) compared to those from high-incidence countries (median year: 1991, IQR:1982–1995), reflecting changes in migration patterns and representing a birth cohort effect. Overall, even within this sub-group of persons from medium-to-low incidence countries there are groups with distinguishable characteristics.

## Discussion

The present study utilized two large cohorts–an immigration cohort with data collected from more than 300,000 individuals, and a genotyping cohort representing 2,290 active TB cases–to investigate the relationship between birth country TB incidence and other immigration-related factors for persons born outside Canada, and TB epidemiology in BC. We found that emigrating from a high-incidence country was associated with increased rates of TB disease, but a decreased likelihood of being involved in a local transmission cluster in BC as compared to individuals emigrating from medium-to-low incidence countries. Our results further suggested that, more recent migrants from high-incidence countries largely represented LTBI reactivation, whereas, long-time residents of Canada from lower incidence regions represent two distinct groups: (i) LTBI reactivation likely related to advanced age, from infections acquired prior to emigration in an era where TB was more prevalent in countries now considered low incidence [28], and (ii) those who have acquired TB locally–as determined by genotypic clustering and risk factors similar to those typically associated with local transmission in low-incidence settings [29,30]. Other low-incidence countries have similarly noted differences in TB rates and likelihood of local transmission according to length of time since immigration [31,32].

Our findings thus underscore the important notion that persons born outside Canada are not a homogeneous group when it comes to local transmission or risk factors. Lack of available data may limit the ability to report more detailed demographic data in many surveillance and population-based studies, particularly an inability to disaggregate data on diverse groups of people [13,33]. But given the goal of surveillance or research is an accurate understanding of TB epidemiology, our results highlight that it is important to report findings at a more granular level to obtain an unbiased picture. This study demonstrated in large, representative, population-based samples that describing people as “foreign-born” is clearly an oversimplification and could lead to bias in our understanding of TB epidemiology and overestimation of local transmission of imported TB. At a minimum, splitting persons born outside of Canada into two groups–people born in high-incidence countries and people born in medium-to-low-incidence countries, will more accurately capture true TB risk.

### Strengths and limitations

A major strength of the present study was the availability of data from large retrospective cohorts spanning multiple years. Yet it should be noted that this study was limited to a single, albeit large and diverse, Canadian province and our results may not be generalizable to all low-incidence settings. Further analysis within the background of other TB risk factors, such as socioeconomic status, may provide additional insight into the influence of birth country on TB rates and patterns of transmission; however, these additional risk factor data were not available for the present cohorts. Additionally, the use of genotyping to understand local transmission has inherent limitations. First, genotyping requires an *Mtb* isolate, and thereby excludes culture-negative cases–approximately 20% of TB diagnoses in BC [15]. However, these often represent extrapulmonary TB cases, which are seen more frequently in individuals born outside a low-incidence country, and both of these factors decrease the likelihood of involvement in a local transmission chain and having an *Mtb* isolate which belongs to a genotype cluster [34–36]. Second, genotyping by MIRU-VNTR examines only 24 loci across the genome and may not provide the discriminatory power necessary to identify true local transmission clusters, although this is unlikely to affect the conclusions of the present study as whole genome sequencing studies have supported MIRU-VNTR clusters representing transmission amongst locally born persons [37,38] and conversely has demonstrated that MIRU-VNTR clusters of *Mtb* isolates from immigrants overestimates local transmission [39,40], which aligns with the epidemiological data of our MIRU-VNTR clusters.

### The problem with using “foreign-born” as a risk factor

Canada’s population, like many low-incidence TB countries, is widely diverse and represents a multicultural mosaic, welcoming >250,000 permanent residents from ~200 countries in 2015 [41]. More than half of new permanent residents come from ten countries, where TB incidence ranges from low (USA, France, UK) to high (Philippines, India, China, Pakistan, Nigeria) and those in between (Iran, Syria) [41]. As the second-largest immigrant receiving province, many people settle in BC [42]. Recognizing the diversity within migrant populations to low incidence countries such as Canada and tailoring interventions to those at the highest risk for TB reactivation is clearly important; however, there is a fine balance between describing higher “risk” groups and developing targeted interventions while not reinforcing stigma. Stigma and concerns about immigration status amongst newcomers to Canada may delay individuals from seeking treatment [6]. Furthermore, the media’s portrayal of migrants as a potential public health risk–bringing diseases such as TB and spreading them to local populations–contributes to this stigma [43,44]. In reality, data from molecular epidemiology studies show that immigrants to low-incidence TB countries who are subsequently diagnosed with TB rarely transmit TB outside of their immediate family [7,10,45]. Therefore, reporting genotypic clustering, which is often a proxy for TB transmission, without considering birth country incidence and other demographic factors can inflate the proportion of local transmission attributable to foreign birth, inaccurately portraying the risk to the public of TB spread from imported disease.

## Conclusion

TB surveillance data and population research provide the evidence upon which many public health programs and policies are based [46]. Substantial evidence will be needed to define the optimal strategy to achieve TB elimination, but a first step towards this is for TB surveillance programs and population studies to recognize the diversity amongst people with TB and to analyze data with the understanding that people born abroad represent populations with variable risks of TB disease. Categorizing persons born outside Canada beyond simply “foreign-born”, particularly in the context of TB epidemiologic and molecular data, is necessary for a more accurate understanding of TB rates and patterns of transmission in low incidence regions.

## Supporting information

S1 Table Characteristics of persons arriving in Canada ≥10 years prior to tuberculosis diagnosis, categorized by birthplace incidence and genotype clustering status. (PDF)

## References

[1] Rossi C, Zwerling A, Thibert L, Rivest P, McIntosh F, Behr MA, et al Mycobacterium tuberculosis transmission over an 11-year period in a low-incidence, urban setting. Int J Tuberc Lung Dis. 2012;16: 312–318. 10.5588/ijtld.11.0204 22230764

[2] Yuen CM, Kammerer JS, Marks K, Navin TR, France AM. Recent Transmission of Tuberculosis—United States, 2011–2014. Sreevatsan S, editor. PLOS ONE. 2016;11: e0153728 10.1371/journal.pone.0153728 27082644 PMC4833321

[3] Stewart RJ, Tsang CA, Pratt RH, Price SF, Langer AJ. Tuberculosis—United States, 2017. MMWR Morb Mortal Wkly Rep. 2018;67: 317–323. 10.15585/mmwr.mm6711a2 29565838 PMC5868206

[4] Story A, Murad S, Roberts W, Verheyen M, Hayward AC, for the London Tuberculosis Nurses Network. Tuberculosis in London: the importance of homelessness, problem drug use and prison. Thorax. 2007;62: 667–671. 10.1136/thx.2006.065409 17289861 PMC2117290

[5] Mulder C, Klinkenberg E, Manissero D. Effectiveness of tuberculosis contact tracing among migrants and the foreign-born population. Eurosurveillance. 2009;14: 19153 10.2807/ese.14.11.19153-en 19317977

[6] Lönnroth K, Mor Z, Erkens C, Bruchfeld J, Nathavitharana RR, van der Werf MJ, et al Tuberculosis in migrants in low-incidence countries: epidemiology and intervention entry points. The International Journal of Tuberculosis and Lung Disease. 2017;21: 624–636. 10.5588/ijtld.16.0845 28482956

[7] Pareek M, Greenaway C, Noori T, Munoz J, Zenner D. The impact of migration on tuberculosis epidemiology and control in high-income countries: a review. BMC Medicine. 2016;14 10.1186/s12916-016-0557-y 27004556 PMC4804514

[8] World Health Organization. Framework towards tuberculosis elimination in low-incidence countries [Internet]. Geneva, Switzerland: World Health Organization; 2014 Available: http://www.who.int/tb/publications/elimination_framework/en/25473715

[9] Vachon J, Gallant V, Siu W. Tuberculosis in Canada, 2016 [Internet]. Public Health Agency of Canada; 2018 Jan. Report No.: Volume 44-3/4. Available: https://www.canada.ca/en/public-health/services/reports-publications/canada-communicable-disease-report-ccdr/monthly-issue/2018-44/issue-3-4-march-1-2018/article-1-tuberculosis-2016.html?utm_source=lyris&utm_medium=email_en&utm_content=1&utm_campaign=ccdr_18-44-3-4 10.14745/ccdr.v44i34a01 PMC6449093 31007614

[10] Khan K, Hirji MM, Miniota J, Hu W, Wang J, Gardam M, et al Domestic impact of tuberculosis screening among new immigrants to Ontario, Canada. Canadian Medical Association Journal. 2015;187: E473–E481. 10.1503/cmaj.150011 26416993 PMC4627893

[11] Liu Y, Weinberg MS, Ortega LS, Painter JA, Maloney SA. Overseas Screening for Tuberculosis in U.S.-Bound Immigrants and Refugees. New England Journal of Medicine. 2009;360: 2406–2415. 10.1056/NEJMoa0809497 19494216

[12] Aldridge RW, Zenner D, White PJ, Williamson EJ, Muzyamba MC, Dhavan P, et al Tuberculosis in migrants moving from high-incidence to low-incidence countries: a population-based cohort study of 519 955 migrants screened before entry to England, Wales, and Northern Ireland. The Lancet. 2016;388: 2510–2518. 10.1016/S0140-6736(16)31008-X 27742165 PMC5121129

[13] Pareek M, Baussano I, Abubakar I, Dye C, Lalvani A. Evaluation of Immigrant Tuberculosis Screening in Industrialized Countries. Emerging Infectious Diseases. 2012;18: 1422–1429. 10.3201/eid1809.120128 22931959 PMC3437731

[14] Public Health Agency of Canada and Canadian Lung Association/Canadian Thoracic Society. Canadian Tuberculosis Standards - 7th edition [Internet]. 2014 [cited 30 Sep 2018]. Available: https://www.canada.ca/en/public-health/services/infectious-diseases/canadian-tuberculosis-standards-7th-edition/edition-22.html

[15] Guthrie JL, Kong C, Roth D, Jorgensen D, Rodrigues M, Hoang L, et al Molecular Epidemiology of Tuberculosis in British Columbia, Canada: A 10-Year Retrospective Study. Clinical Infectious Diseases. 2017;66: 849–856. 10.1093/cid/cix906 29069284 PMC5850024

[16] Ronald LA, Campbell JR, Balshaw RF, Romanowski K, Roth DZ, Marra F, et al Demographic predictors of active tuberculosis in people migrating to British Columbia, Canada: a retrospective cohort study. CMAJ. 2018;190: E209–E216. 10.1503/cmaj.170817 29483329 PMC5826706

[17] BC Communicable Disease Policy Advisory Committee. BC strategic plan for tuberculosis prevention, treatment, and control [Internet]. 2012 Jun. Available: http://www.bccdc.ca/NR/rdonlyres/371821DC-D135-4BC6-8AD9-4F09CF667B29/0/BC_Strategic_Plan_Tuberculosis.pdf

[18] BC Centre for Disease Control [creator]. (2015): BC Provincial TB Registry (BCCDC-iPHIS). Population Data BC [publisher]. Data Extract. BCCDC (2014) [Internet]. Available: http://www.popdata.bc.ca/data

[19] British Columbia Ministry of Health [creator]. (2015): Consolidation File (MSP Registration & Premium Billing). V2. Population Data BC [publisher]. Data Extract. MOH (2014) [Internet]. Available: http://www.popdata.bc.ca/data

[20] Citizenship and Immigration Canada [creator]. (2014): CIC Permanent Residents File. Population Data BC [publisher]. Data Extract. CIC (2015) [Internet]. Available: http://www.popdata.bc.ca/data

[21] France AM, Grant J, Kammerer JS, Navin TR. A Field-Validated Approach Using Surveillance and Genotyping Data to Estimate Tuberculosis Attributable to Recent Transmission in the United States. Am J Epidemiol. 2015;182: 799–807. 10.1093/aje/kwv121 26464470 PMC5996379

[22] Lambregts-van Weezenbeek CSB, Sebek MMGG, van Gerven PJHJ, de Vries G, Verver S, Kalisvaart NA, et al Tuberculosis contact investigation and DNA fingerprint surveillance in The Netherlands: 6 years’ experience with nation-wide cluster feedback and cluster monitoring. The International Journal of Tuberculosis and Lung Disease. 2003;7: S463–S470. 14677839

[23] Sintchenko V, Gilbert GL. Utility of genotyping of Mycobacterium tuberculosis in the contact investigation: A decision analysis. Tuberculosis. 2007;87: 176–184. 10.1016/j.tube.2006.10.003 17161653

[24] Kamper-Jørgensen Z, Andersen AB, Kok-Jensen A, Bygbjerg IC, Andersen PH, Thomsen VO, et al Clustered Tuberculosis in a Low-Burden Country: Nationwide Genotyping through 15 Years. J Clin Microbiol. 2012;50: 2660–2667. 10.1128/JCM.06358-11 22675129 PMC3421533

[25] Globan M, Lavender C, Leslie D, Brown L, Denholm J, Raios K, et al Molecular epidemiology of tuberculosis in Victoria, Australia, reveals low level of transmission. The International Journal of Tuberculosis and Lung Disease. 2016;20: 652–658. 10.5588/ijtld.15.0437 27084820

[26] World Health Organization. Tuberculosis country profiles [Internet]. [cited 8 Jul 2018]. Available: http://www.who.int/tb/country/data/profiles/en/

[27] Tsang CA, Langer AJ, Navin TR, Armstrong LR. Tuberculosis Among Foreign-Born Persons Diagnosed ≥10 Years After Arrival in the United States, 2010–2015. American Journal of Transplantation. 2017;17: 1414–1417. 10.1111/ajt.14300 28333913 PMC5657888

[28] Schaaf HS, Collins A, Bekker A, Davies PDO. Tuberculosis at extremes of age. Respirology. 2010;15: 747–763. 10.1111/j.1440-1843.2010.01784.x 20546192

[29] Nava-Aguilera E, Andersson N, Harris E, Mitchell S, Hamel C, Shea B, et al Risk factors associated with recent transmission of tuberculosis: systematic review and meta-analysis. Int J Tuberc Lung Dis. 2009;13: 17–26. 19105874

[30] Adam HJ, Guthrie JL, Bolotin S, Alexander DC, Stuart R, Pyskir D, et al Genotypic characterization of tuberculosis transmission within Toronto’s under-housed population, 1997–2008. The International Journal of Tuberculosis and Lung Disease. 2010;14: 1350–1353. 20843430

[31] Vos AM, Meima A, Verver S, Looman CWN, Bos V, Borgdorff MW, et al High Incidence of Pulmonary Tuberculosis a Decade after Immigration, Netherlands. Emerg Infect Dis. 2004;10: 736–739. 10.3201/eid1004.030530 15200873 PMC3323101

[32] Séraphin MN, Lauzardo M. Mycobacterium tuberculosis complex transmission is not associated with recent immigration (≤5years) in Florida. Infection, Genetics and Evolution. 2015;36: 547–551. 10.1016/j.meegid.2015.08.038 26325684

[33] Hargreaves S, Carballo M, Friedland JS. Screening migrants for tuberculosis: where next? The Lancet Infectious Diseases. 2009;9: 139–140. 10.1016/S1473-3099(09)70026-X 19246015

[34] Torgersen J, Dorman SE, Baruch N, Hooper N, Cronin W. Molecular Epidemiology of Pleural and Other Extrapulmonary Tuberculosis: A Maryland State Review. Clin Infect Dis. 2006;42: 1375–1382. 10.1086/503421 16619148

[35] Lillebaek T, Andersen ÅB, Dirksen A, Smith E, Skovgaard LT, Kok-Jensen A. Persistent High Incidence of Tuberculosis in Immigrants in a Low-Incidence Country. Emerg Infect Dis. 2002;8: 679–684. 10.3201/eid0807.010482 12095434 PMC2730343

[36] Al-Nakeeb Z, Gupta V, Bell C, Woodhead M. Are we missing opportunities to confirm the diagnosis of tuberculosis by microbial culture? Respiratory Medicine. 2013;107: 2022–2028. 10.1016/j.rmed.2013.09.016 24140285

[37] Gardy JL, Johnston JC, Ho Sui SJ, Cook VJ, Shah L, Brodkin E, et al Whole-genome sequencing and social-network analysis of a tuberculosis outbreak. N Engl J Med. 2011;364: 730–739. 10.1056/NEJMoa1003176 21345102

[38] Stucki D, Ballif M, Bodmer T, Coscolla M, Maurer A-M, Droz S, et al Tracking a tuberculosis outbreak over 21 years: strain-specific single-nucleotide polymorphism typing combined with targeted whole-genome sequencing. J Infect Dis. 2015;211: 1306–1316. 10.1093/infdis/jiu601 25362193 PMC4447836

[39] Stucki D, Ballif M, Egger M, Furrer H, Altpeter E, Battegay M, et al Standard Genotyping Overestimates Transmission of Mycobacterium tuberculosis among Immigrants in a Low-Incidence Country. J Clin Microbiol. 2016;54: 1862–1870. 10.1128/JCM.00126-16 27194683 PMC4922098

[40] Jamieson FB, Teatero S, Guthrie JL, Neemuchwala A, Fittipaldi N, Mehaffy C. Whole-genome sequencing of the Mycobacterium tuberculosis Manila sublineage results in less clustering and better resolution than mycobacterial interspersed repetitive-unit-variable-number tandem-repeat (MIRU-VNTR) typing and spoligotyping. J Clin Microbiol. 2014;52: 3795–3798. 10.1128/JCM.01726-14 25078914 PMC4187780

[41] Government of Canada. Annual Report to Parliament on Immigration, 2016 [Internet]. 31 Oct 2016 [cited 3 Jun 2018]. Available: https://www.canada.ca/en/immigration-refugees-citizenship/corporate/publications-manuals/annual-report-parliament-immigration-2016.html#abimm

[42] Statistics Canada. Canadian Demographics at a Glance, Second edition [Internet]. 19 Feb 2016 [cited 16 Jun 2018]. Available: http://www.statcan.gc.ca/pub/91-003-x/91-003-x2014001-eng.htm

[43] Littleton J, Park J, Thornley C, Anderson A, Lawrence J. Migrants and tuberculosis: analysing epidemiological data with ethnography. Australian and New Zealand Journal of Public Health. 2008;32: 142–149. 10.1111/j.1753-6405.2008.00191.x 18412685

[44] Reitmanova S, Gustafson DL. Exploring the Mutual Constitution of Racializing and Medicalizing Discourses of Immigrant Tuberculosis in the Canadian Press. Qualitative Health Research. 2012;22: 911–920. 10.1177/1049732312441087 22427457

[45] Kunimoto D, Sutherland K, Wooldrage K, Fanning A, Chui L, Manfreda J, et al Transmission characteristics of tuberculosis in the foreign-born and the Canadian-born populations of Alberta, Canada. The International Journal of Tuberculosis and Lung Disease. 2004;8: 1213–1220. 15527153

[46] Canada, Health Canada. Health Canada’s strategy against tuberculosis for First Nations on-reserve. 2012.

