# Supplementary material for: The problem with defining foreign birth as a risk factor in tuberculosis epidemiology studies
Source: PLoS One. 2019 Apr 30;14(4):e0216271. doi: 10.1371/journal.pone.0216271 (PMC6490926; doi:10.1371/journal.pone.0216271)
Supplement: S1 Table — (PDF) [file pone.0216271.s001.pdf]

S1 Table. Characteristics of persons arriving in Canada ≥10 years prior to tuberculosis diagnosis, categorized by birthplace incidence and genotype clustering status.

	High-Incidence		Medium-to-low Incidence	
Characteristics	Clustered	Unique	Clustered	Unique
Total — *n*	249	606	22	59
Male — *n* (%)	146 (58.6)	350 (57.8)	17 (77.3)	31 (52.5)
Age at immigration — median (IQR)	39 (26–56)	42 (28–57)	9 (4–26)	30 (21–40)
Age at diagnosis — median (IQR)	63 (49–77)	68 (50–79)	53 (46–60)	75 (63–82)
Year of arrival — median (IQR)	1991 (1982–1996)	1990 (1982–1994)	1966 (1957–1988)	1968 (1957–1982)
Region of birth				
Asia	237 (95.2)	575 (94.9)	1 (4.5)	9 (15.3)
United States/Europe*	0 (0.0)	0 (0.0)	15 (68.2)	31 (52.5)
Other	12 (4.8)	31 (5.1)	6 (27.3)	19 (32.2)

Abbreviations: IQR, interquartile range.
*Eastern Europe excluded.
